# Personalia

**Published:** 1914-09-19

**Authors:** 


### Personalia.

Mr. J. T. H. Madill has resigned his appointment as fifth assistant medical officer at Hanwell Mental Hospital.

Dr. Wm. Campbell has resigned his post under the Bradford Corporation at the Leeds Road Hospital, in order to take up an appointment at Edinburgh University.

A further member of the honorary staff of the Leicester Royal Infirmary, in the person of Major Sevestre, has been called up for duty in connection with the 5th Northern General Hospital.

Mr. Geo. Wherry, M.C. Cantab., M.R.C.S. Eng., senior hon. surgeon at Addenbrooke's Hospital, who left Cambridge about July 26, 1914, for a holiday in the Alps, ultimately made his way to Genoa and returned by the White Star Line.

Major Ernest Gray has been appointed chairman, and Mr. Cyril Cobb vice-chairman, of the Special Committee which the London County Council has appointed to make arrangements for the establishment of its Ambulance Service.

Mr. T. H. F. Lapthorn, who has succeeded the late Mr. J. J. Young as chairman of the committee of management of the Royal Portsmouth and Gosport Hospital, thus vacates the vice-chairmanship by this promotion. His successor in the latter office is Mr. George Cooke, J.P., whose association with the hospital has been a long one, and it is hoped that, with his experience on behalf of the Eye and Ear Infirmary, he may be able to increase the support from Gosport. For Gosport is asserted to receive a sovereign's worth of treatment for every ten shillings which it subscribes, although a good many legacies have been received from this quarter.

